# Beyond Imaging Overuse: Reframing Appropriateness as a Framework for Value-Based and Sustainable Radiology

**DOI:** 10.3390/diagnostics16213667

**Published:** 2026-11-06

**Authors:** Serena Carriero, Jacopo D’Argenzio, Salvatore Alessio Angileri, Pierpaolo Biondetti, Rossella Catona, Anna Colarieti, Letizia Di Meglio, Chloe Yeabin Jung, Carolina Lanza, Luca Nicosia, Giuseppe Pellegrino, Filippo Pesapane, Jacopo Pozzi, Gianpaolo Carrafiello

**Affiliations:** 1 Radiology and Interventional Radiology, Fondazione IRCCS Ca’ Granda Ospedale Maggiore Policlinico, Via Francesco Sforza 35, 20122 Milan, Italy; serena.carriero@gmail.com (S.C.); alessioangileri@gmail.com (S.A.A.); pierpaolo.biondetti@policlinico.mi.it (P.B.); letizia.dimeglio@policlinico.mi.it (L.D.M.); carolina.lanza@unimi.it (C.L.); gianpaolo.carrafiello@unimi.it (G.C.); 2 Postgraduate School in Radiodiagnostics, Università degli Studi di Milano, Via Festa del Perdono 7, 20122 Milan, Italy; rossella.catona@unimi.it (R.C.); yeabin.jung@unimi.it (C.Y.J.); jacopo.pozzi@unimi.it (J.P.); 3 Department of Translational Medicine, University of Eastern Piemonte UPO, Via Solaroli n.17, 28100 Novara, Italy; anna.colarieti@uniupo.it; 4 Division of Breast Radiology, Department of Medical Imaging and Radiation Sciences, IEO European Institute of Oncology IRCCS, Via Ripamonti 435, 20141 Milan, Italy; luca.nicosia@ieo.it (L.N.); filippo.pesapane@ieo.it (F.P.); 5 Department of Oncology and Hemato-Oncology, Università degli Studi di Milano, Via Festa del Perdono 7, 20122 Milan, Italy; giuseppe.pellegrino@unimi.it; 6 Unit of Diagnostic Imaging and Stereotactic Radiosurgery, CDI Centro Diagnostico Italiano S.p.A., Via Simone Saint Bon 20, 20147 Milan, Italy

**Keywords:** imaging appropriateness, low-value imaging, diagnostic stewardship, clinical decision support, radiology sustainability

## Abstract

Diagnostic imaging is central to contemporary healthcare, but increasing demand has intensified concerns regarding inappropriate and low-value examinations. Imaging appropriateness should be understood as a multidimensional quality framework rather than a simple reduction in examination volume. This narrative review synthesizes evidence on clinical outcomes, radiation protection, healthcare costs, organizational efficiency, equity, incidental findings, and environmental sustainability. A structured PubMed search was complemented by targeted database and institutional searches and selective citation tracking. Low-value imaging may disrupt diagnostic pathways through diagnostic uncertainty, unnecessary follow-up, avoidable radiation exposure, additional costs, waiting-list pressure, and environmental burden. Mitigation strategies include referral guidelines, clinical decision support, radiologist-led vetting, standardized incidental findings management, and audit and feedback. Their effects vary by setting and outcome: improved referral scores do not necessarily reduce utilization or improve patient outcomes. Environmental estimates depend on imaging modalities, electricity supply, and assessment boundaries, while proposed artificial intelligence applications require prospective validation. We translate this evidence into an operational pathway linking examination justification, modality and protocol selection, reporting, follow-up, and service evaluation. Imaging appropriateness therefore requires reducing low-value examinations while preserving diagnostic safety and timely, equitable access to clinically beneficial imaging.

## 1. Introduction

In modern medicine, diagnostic imaging has evolved from a secondary tool into a structural component of the patient care pathway. Computed tomography (CT), magnetic resonance imaging (MRI), ultrasound, conventional radiology, and nuclear medicine are indispensable for diagnosis, staging, follow-up, prognostic stratification, therapeutic monitoring, and emergency management. This growing centrality is reflected in utilization trends: a study of 16 Organisation for Economic Co-operation and Development (OECD) countries between 2011 and 2020 confirmed a widespread increase in imaging use, with significant variability between health systems and relevant clinical, organizational, and economic implications [1,2].

Imaging workload also depends on the number and complexity of images within each examination. A retrospective study of a tertiary centre documented increasing radiographic, CT, and MRI activity between 2005 and 2020 [3]. In a separate institutional study covering 1999–2010, the number of CT and MRI images requiring interpretation per minute per radiologist increased from 2.9 to 16.1 after adjustment for staffing [4]. These findings illustrate why examination counts alone incompletely represent reporting demand.

Artificial intelligence (AI) is frequently proposed as a response to increasing diagnostic demand, with applications in triage, detection, prioritization, reporting, decision support, and workflow optimization [5,6,7,8]. Rather than automatically mitigating the “data deluge”, however, emerging evidence suggests that AI may also introduce additional validation, integration, and post-processing requirements and therefore does not necessarily reduce workload in routine practice [5,7,9].

Several related terms require distinction. Imaging overuse denotes testing beyond a level expected to improve patient care. Inappropriate imaging denotes a mismatch between the examination and the clinical indication, patient context, modality, timing, or evidence-based referral criteria; it is not established by a negative result alone [10,11]. The choice of eligible population and examination interval also matters, as illustrated by screening guidance [12,13]. Low-value imaging describes a poor balance between expected clinical benefit and potential harms or resource use [14,15,16]. These categories overlap, but they are not interchangeable: guideline discordance is a process measure, whereas value concerns consequences for patients and the health system. Diagnostic stewardship is the coordinated management of test selection, performance, interpretation, and follow-up to improve that balance [10,17].

The causes underlying inappropriateness and low-value imaging are multifactorial, arising from clinical uncertainty, organizational pressures, cultural factors, medico-legal concerns, financial incentives, self-referral practices, referring-physician behavior, and patient expectations [17,18,19,20]. Defensive medicine is one relevant contributor, with imaging sometimes requested primarily to reduce perceived medico-legal risk rather than because it is expected to change management; observational and policy literature has linked this behavior to avoidable imaging use, cost, and radiation exposure [18,21,22].

The consequences of inappropriate imaging extend beyond the unnecessary examination itself. At the same time, findings discovered outside the primary clinical question are not intrinsically low value: opportunistic CT assessment and incidental observations may provide prognostic or metabolic information when they are validated and clinically actionable [23,24,25,26]. The problem arises when unexpected findings are indeterminate or managed outside standardized pathways, potentially triggering repeated imaging, specialist referrals, invasive procedures, and diagnostic cascades [27,28,29].

Moreover, overuse increases cumulative radiation exposure, particularly in younger and repeatedly imaged populations, and may generate patient anxiety through unnecessary follow-up [10,17,30,31]. These clinical and psychological costs are compounded by an increasingly relevant sustainability dimension, since unnecessary imaging may generate avoidable energy use, contrast-media consumption, consumables, data-storage requirements, and carbon emissions [32,33,34].

**Figure 1:**
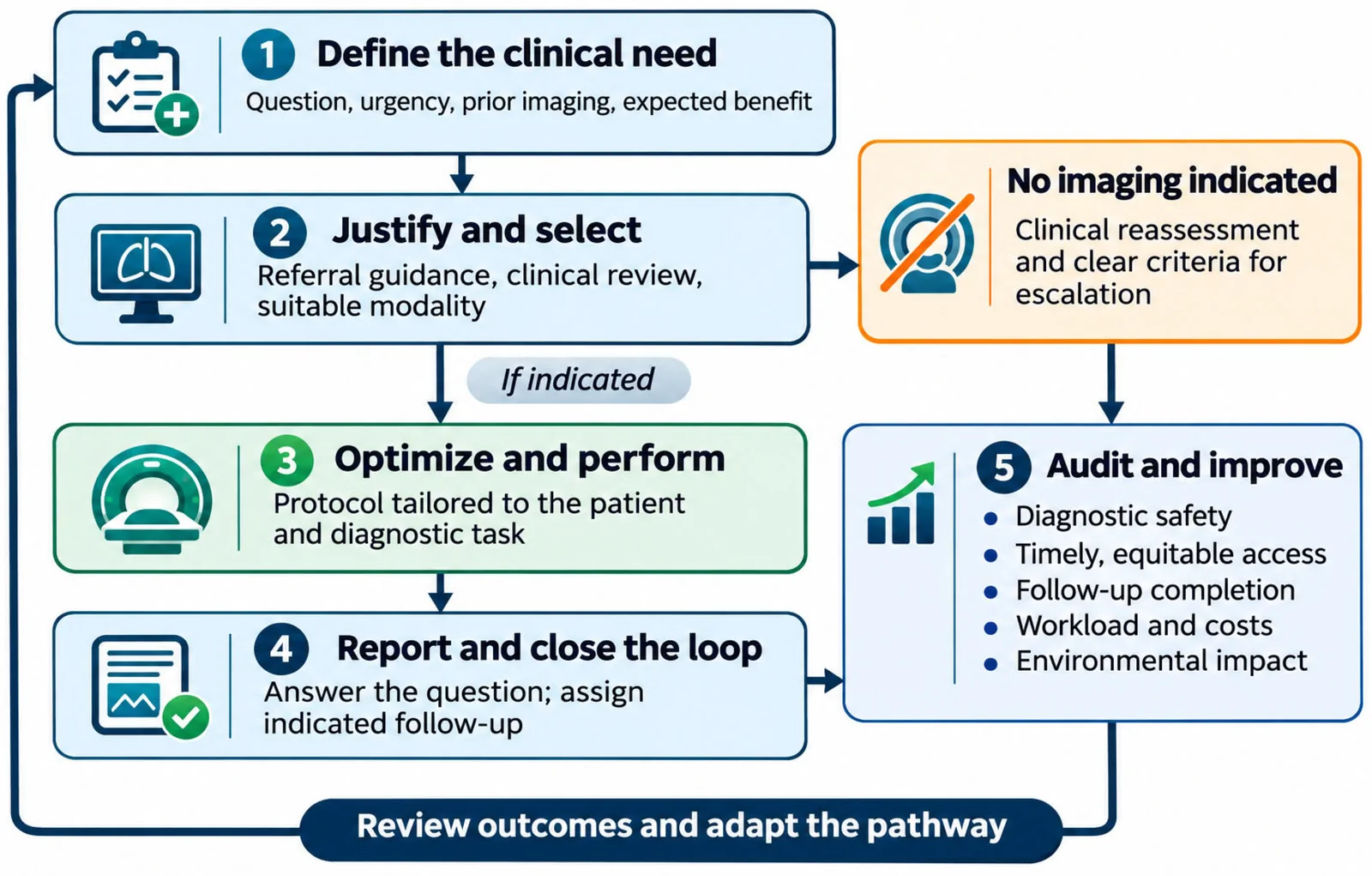
Proposed pathway for imaging appropriateness across the patient journey. The decision to image is revisited through justification, protocol selection, reporting, follow-up, and audit. Clinical follow-up remains necessary when imaging is not indicated. The feedback loop includes patient safety and equitable access alongside utilization, costs, and environmental impact. This is an evidence-informed operational proposal, not a validated decision rule.

This narrative review synthesizes evidence on imaging appropriateness across clinical, radiation-protection, economic, organizational, and environmental domains, and examines tools used before and after imaging acquisition. Previous reviews have characterized low-value examinations, evaluated interventions and costs, or addressed environmental sustainability [14,17,32,35,36]. Our contribution is to connect these domains across the patient pathway, linking examination justification and protocol selection with post-imaging management and service-level evaluation. The proposed framework (Figure 1) combines established approaches with explicit responsibilities and measurable indicators, including diagnostic safety, timely access, and equity. It is intended to guide implementation and evaluation rather than provide a validated appropriateness score.

## 2. Methods

### 2.1. Narrative Review Design

This narrative review integrates original studies, evidence syntheses, professional guidance, and policy documents relevant to imaging appropriateness. Sources were selected purposively for their relevance to the clinical, economic, organizational, or environmental questions addressed. Priority was given to clearly defined outcomes, comparative evidence where available, clinical applicability, and authoritative guidance. Studies of specific indications or technologies were included when they illustrated a broader mechanism or an important limitation. Given the narrative design, the Preferred Reporting Items for Systematic Reviews and Meta-Analyses (PRISMA) framework was not applied, and selection did not follow a systematic sequence of title/abstract screening and full-text eligibility assessment. No meta-analysis or formal risk-of-bias scoring was undertaken.

### 2.2. Literature Search

A structured PubMed search was conducted on 29 April 2026 using the following search string:

*((“low-value imaging”[Title/Abstract] OR “inappropriate imaging”[Title/Abstract] OR “unnecessary imaging”[Title/Abstract] OR “imaging overuse”[Title/Abstract] OR “diagnostic stewardship”[Title/Abstract]) AND (“diagnostic imaging”[Title/Abstract] OR radiology[Title/Abstract] OR “computed tomography”[Title/Abstract] OR CT[Title/Abstract] OR MRI[Title/Abstract] OR “magnetic resonance imaging”[Title/Abstract]) AND (appropriateness[Title/Abstract] OR “appropriate use”[Title/Abstract] OR “clinical decision support”[Title/Abstract] OR “radiation exposure”[Title/Abstract] OR cost[Title/Abstract] OR economic[Title/Abstract] OR “healthcare utilization”[Title/Abstract] OR sustainability[Title/Abstract])).*

The search yielded 172 records, which were assessed by two authors through a purposive, non-systematic selection process. Selection was guided by thematic relevance, methodological characteristics, clinical applicability, and the contribution of individual studies to the review objectives. Priority was given to publications reporting clearly defined outcomes, comparative evidence where available, or findings applicable beyond narrowly defined clinical scenarios. This process was intended to identify informative evidence for narrative synthesis rather than to undertake exhaustive eligibility assessment according to a systematic-review protocol.

Of the 172 records identified, 23 were retained and cited in the final manuscript. An additional 130 references were identified through complementary targeted searches, professional and institutional guidance, and citation tracking, as described below, resulting in a final bibliography of 153 sources.

### 2.3. Identification of Guidelines, Policy Documents, and Decision-Support Tools

Following the initial PubMed search, complementary topic-specific searches were conducted in PubMed, Scopus, and Embase during manuscript preparation, before the original submission in October 2026. These searches addressed imaging appropriateness, referral guidelines, clinical decision support, incidental findings, radiation justification, imaging costs, waiting times, and environmental sustainability. Search concepts were adapted to the respective thematic domains rather than combined into a single additional search strategy. Selective backward and forward citation tracking was also undertaken to identify relevant publications. Institutional webpages cited in the original manuscript were accessed on 10 August 2026.

Institutional sources included the American College of Radiology (ACR; acr.org), the European Society of Radiology (ESR; myESR.org), the Royal College of Radiologists (RCR; rcr.ac.uk and irefer.org.uk), and the Italian Society of Medical and Interventional Radiology (SIRM; sirm.org). Materials included the ACR Appropriateness Criteria and decision-support frameworks [37], ACR incidental findings recommendations [38], Fleischner Society pulmonary nodule guidance [39], ESR iGuide methodology [40], RCR iRefer [41], and SIRM appropriateness and radiological-act documents [42,43]. Specialty-specific examples included whole-body MRI in oncology and European breast-imaging recommendations [44,45].

Other sources included Choosing Wisely [15,46], EUR-Lex (eur-lex.europa.eu) [47], and the EU Publications Office (op.europa.eu) [48]. Documents were selected for the authority of the issuing body, an explicit scope or intended population, and relevance to an imaging decision. Tool descriptions informed the account of functionality, while effectiveness was assessed from clinical evaluations.

### 2.4. Complementary Reference Selection

Complementary literature identification continued throughout manuscript preparation, with targeted searches and citation tracking undertaken before the original submission in October 2026. During revision, additional targeted PubMed and publisher searches and citation checks were extended through 1 November 2026, particularly for artificial intelligence, environmental sustainability, referral vetting, and equity in imaging access. This approach allowed relevant recent developments to be incorporated while preserving the initial structured PubMed search as the bibliographic foundation of the review. These complementary activities remained purposive and non-systematic and were not intended to establish exhaustive literature coverage.

Bibliographic duplicates were identified by DOI, PubMed identifier where available, and title. Related reports were not treated as interchangeable publications, and overlapping evidence was considered jointly. For example, the MIDAS trial, its abdominal-imaging analysis, and its implementation report were interpreted as complementary reports rather than independent replications [49,50,51]. Critical interpretation considered study design, comparator, setting, outcome definition, and the distinction between process measures and patient outcomes.

Generative AI (ChatGPT/Codex, OpenAI) was used to assist with the preparation of the conceptual illustration presented in Figure 1. The final graphical content and its scientific interpretation were reviewed and approved by the authors.

## 3. Discussion

Imaging overuse can affect patient safety, resource allocation, service capacity, and environmental sustainability [10,32,35,52]. Table 1 summarizes the main mechanisms and corresponding actions. Table 2 places selected quantitative findings alongside their design limitations; the estimates have different denominators and should not be treated as a single prevalence or pooled intervention effect.

### 3.1. Clinical Impact and Diagnostic Pathway Disruption

An examination should be evaluated against the clinical question, pre-test probability, and expected effect on management, rather than against examination volume alone [10,36,52,59].

A key distinction must be made between diagnostic yield and clinical usefulness. A negative examination is not intrinsically low value; on the contrary, it may be highly appropriate when it reliably excludes a clinically relevant condition or reduces diagnostic uncertainty. The problem arises when imaging is performed in contexts of low pre-test probability or poorly defined clinical questions, where the expected contribution to decision-making is minimal and the potential to improve patient-relevant outcomes is limited [36,60].

When poorly indicated imaging generates ambiguous findings, it may add uncertainty and downstream investigations without a commensurate clinical benefit [17,60].

Incidental findings represent a paradigmatic mechanism through which this process may occur. Incidental findings are common and are not inherently harmful; some may provide clinically useful information. However, when their significance is uncertain or their management is not standardized, they may trigger diagnostic cascades characterized by repeated imaging, specialist referrals, patient anxiety, and occasionally invasive procedures [27,28,29,38,61]. Structured recommendations, such as those developed by the Fleischner Society for incidental pulmonary nodules, illustrate how risk-stratified follow-up can reduce unnecessary downstream testing while preserving clinically meaningful surveillance [27,28,29,39].

Clinical examples illustrate both the problem and the limits of current evidence. In an Italian emergency department, 25% of 2498 evaluable head CT requests were classified as usually not appropriate; 410 additional requests could not be assessed because clinical information was insufficient [53]. The denominator therefore excludes a group that may differ systematically from assessable requests. A retrospective external validation of the Emergency CT Head Score found high sensitivity for CT abnormalities, but did not test prospective withholding of imaging or subsequent clinical outcomes [62]. Abdominal-imaging observations from MIDAS likewise illustrate the importance of clinical information and referral criteria [49].

In elective chest CT, deviations from incidental findings guidance can lead to unwarranted follow-up recommendations [63]. Inpatient observations also identify inappropriate examinations and associated resource use [64]. These setting-specific findings support local audit, but do not establish a universal fraction of imaging that can safely be removed.

### 3.2. Radiation Exposure and Radioprotection Implications

The fundamental principle of radiation protection is that justification precedes optimization. Technical advances in dose reduction, while essential, cannot compensate for an examination that is not clinically indicated. This principle is embedded in European regulatory and professional frameworks, which identify justification as the primary safeguard against unnecessary medical exposure [47,48,65].

Radiation risk must be considered both individually and cumulatively. Repeated imaging, non-essential follow-up, and serial investigations contribute to aggregate exposure over time, and inappropriate imaging therefore contributes directly to avoidable radiation burden [66,67,68,69,70].

Radiation exposure warrants particular attention in children, young adults, and repeatedly imaged patients [71,72,73,74,75,76,77]. Cohort studies have reported associations between CT exposure and subsequent malignancy, including a dose–response relationship for brain cancer in EPI-CT [72,73,74]. These are observational estimates. Underlying conditions may influence both imaging and cancer risk, and symptoms of an undiagnosed tumour may prompt CT, creating confounding by indication or reverse causation. Exposure reconstruction, missing examinations, latency assumptions, and differences between historical and contemporary protocols also affect interpretation. Lag periods and sensitivity analyses reduce some biases; in EPI-CT, the association persisted when follow-up began more than five years after the first scan [73].

Model-based lifetime projections are a separate evidence category: they combine examination volumes, organ doses, and risk-model assumptions rather than count cancers observed to have been caused by CT [75]. Population associations and projections should inform justification and optimization, but do not determine an individual patient’s risk–benefit balance or justify withholding a clinically necessary examination.

Appropriate protocol selection is therefore important alongside examination justification. Validated dose-optimized protocols can reduce exposure while preserving the required diagnostic task [78], whereas ultrasound or MRI may provide alternatives when they can answer the clinical question with sufficient accuracy and timeliness [48].

AI studies also address different endpoints. Segmentation and radiomics analyses on low- or ultra-low-dose datasets establish task-specific technical feasibility [79,80]. Reconstruction studies have reported improved image quality or opportunities for dose reduction, including phantom and initial clinical evaluations [81,82,83,84]. Performance in a particular dataset or reconstruction task does not establish that dose can be reduced safely across all scanners, indications, or patient groups.

Follow-up strategies represent an additional dimension of radioprotection. Guideline-based management of incidental findings can prevent unnecessary serial imaging, making appropriate follow-up itself a radioprotective intervention [39]. Overall, radioprotection is inseparable from appropriateness: the most effective way to avoid unnecessary radiation is to ensure that each examination is justified within a coherent diagnostic pathway [47,66,70].

### 3.3. Economic Burden and Healthcare Resource Utilization

The economic impact of low-value imaging extends beyond the immediate cost of the examination and includes direct, indirect, and opportunity costs [32,85].

Direct costs include examination expenses, scanner time, personnel, consumables, contrast agents, reporting, and administrative processes. A proportion of these resources is allocated to examinations with limited clinical utility in both outpatient and inpatient settings [54,64,66,85].

Indirect costs arise from downstream consequences, including additional imaging, specialist consultations, invasive procedures, possible procedure-related complications, and management of incidental findings. Diagnostic cascades can substantially amplify initial expenditure, particularly when follow-up is not guideline-concordant [29,60,63,86].

Opportunity costs arise when low-value examinations occupy capacity that could serve patients with stronger indications [52,85]. However, an avoided examination does not automatically release its full tariff as a cash saving. Staffing, equipment, and premises include fixed costs; financial benefit depends on variable costs avoided and whether released capacity is productively redeployed.

Musculoskeletal imaging, low-probability neuroimaging, routine preoperative testing, and unnecessary follow-up are recurrent targets [29,54,87,88,89,90]. In a Massachusetts claims analysis, guideline-discordant imaging accounted for more than 20% of annual imaging expenditure for three specified musculoskeletal indications [54]. This condition-specific estimate cannot be extrapolated to all imaging or healthcare spending. International cost evidence is heterogeneous in currency, perspective, case definition, and included downstream costs [32]. Resource reallocation is a plausible benefit of value-based radiology, but requires local economic and implementation assessment [52,85,91,92].

### 3.4. Waiting Lists and Organizational Efficiency

The relationship between imaging appropriateness and waiting-list pressure can be understood in terms of demand-capacity mismatch and resource displacement. Demand for imaging continues to grow, often outpacing acquisition and reporting capacity [1,3,4]. Inappropriate imaging can exacerbate this imbalance by consuming scanner availability, radiographer workload, radiologist reporting time, and administrative capacity that could otherwise be allocated to clinically justified examinations [52,85].

Overuse and underuse may coexist: low-value examinations can consume capacity while clinically justified investigations remain unavailable or delayed [14,36]. Geographic distance, scanner distribution, transport, insurance or out-of-pocket costs, referral access, and shortages of trained staff can shape that imbalance. International evidence on cancer imaging emphasizes the consequences of unequal access to diagnostic infrastructure and expertise [65]. Within a health system, socioeconomic and institutional factors may also influence modality selection. A United States observational study of children with pyelonephritis associated lower-income residential areas and non-teaching settings with greater use of CT rather than ultrasound among children who were imaged [93]. Such associations identify inequities for investigation, but do not establish that each CT was inappropriate.

An appropriateness programme should therefore track access as well as avoided examinations. Waiting times by urgency and geography, completion of indicated follow-up, and reasons for non-attendance should be examined across relevant population groups. Where guideline-preferred imaging is unavailable, a documented, clinically reasonable alternative or referral pathway is preferable to simple denial. Resource savings should be judged partly by whether they improve timely access to beneficial care.

Addressing this problem requires more than increasing imaging capacity. Improvements in referral quality, triage, prioritization, and pathway design are also essential [40,59]. Systematic evidence suggests that imaging referral guidelines can influence both patient management and radiology-service organization, supporting more efficient allocation of resources [94].

High volumes also contribute to workload and burnout concerns [3,5,9,95]. Referral support and radiologist-led vetting may improve allocation, but evidence of reduced waiting times or diagnostic delays should be measured directly rather than inferred from fewer requests [91].

### 3.5. Environmental Sustainability and Energy Burden

Diagnostic imaging has a measurable environmental footprint involving energy consumption for image acquisition and cooling, contrast media, consumables, equipment maintenance, digital archiving, and patient-related logistics. The scale and composition of this footprint vary by modality and clinical setting and may be particularly relevant in technologically intensive subspecialties [33,34,96,97,98,99,100,101,102].

Cavanagh et al. modeled emissions associated with inappropriate imaging in United States Medicare fee-for-service Part B from 2017 to 2021 [58]. Their estimate combined utilization of MRI, CT, radiography, and ultrasound with inappropriateness assumptions informed by the 2014 Medicare Imaging Demonstration and published emission factors. An assumed 4–26% inappropriateness range yielded an average annual estimate of 3.55–129.2 kilotonnes of carbon dioxide equivalent (CO_2_e). These are low/high modeled estimates, not a confidence interval or directly measured emissions from individually adjudicated inappropriate examinations.

Conceptually, such estimates combine examination counts, the assumed proportion inappropriate, and modality-specific emission factors. Results depend on scanner and protocol characteristics, electricity generation, utilization and standby time, and whether boundaries include equipment manufacture, consumables, data storage, or travel. Attributional life cycle assessment allocates an average footprint to a scan; consequential estimates address the incremental impact of changing activity. In two Australian services, average MRI and CT footprints were 17.5 and 9.2 kg CO_2_e per scan, respectively, whereas the corresponding additional-scan estimates were about 1.1 kg for each when standby emissions were excluded [33]. Thus, cancelling an individual scan while equipment remains powered may save much less than its allocated average footprint.

Avoiding low-value imaging can reduce acquisition-related resource use and prevent downstream cascades, while scheduling and safe equipment power management can address persistent idle consumption [32,33,35,99,100,103,104]. Environmental estimates should state the setting, functional unit, system boundary, and assumptions, with sensitivity analyses where feasible. Comparisons between modalities require equivalent clinical tasks and adequate diagnostic performance; lower emissions alone do not make a less suitable examination appropriate.

Environmental sustainability also involves workforce and financial viability [34,96,105,106,107,108]. Benefits attributed to AI should account for training and inference energy, hardware, storage, and any additional imaging generated by the system. Reduced computation or shorter acquisition is not, by itself, a demonstrated net environmental benefit across the patient pathway.

### 3.6. Tools to Improve Appropriateness Before Imaging Acquisition

#### 3.6.1. Referral Guidelines and Appropriateness Criteria

Referral guidelines and appropriateness criteria translate evidence and expert consensus into practical recommendations for selecting imaging in specific clinical scenarios [94,109,110].

The ACR Appropriateness Criteria represent one of the most structured evidence-based systems and are developed through a formal methodology [109,110]. In Europe, ESR iGuide applies similar principles through integrated decision support at the point of imaging request [111,112]. Additional frameworks include the RCR iRefer guidelines and the Italian SIRM appropriateness model and radiological-act framework, which emphasize both justification and the technical and professional quality of imaging practice [41,42,43]. More broadly, initiatives such as Choosing Wisely have promoted awareness of low-value care and unnecessary imaging [15,46,59].

Despite availability of these resources, an implementation gap persists. Guideline adherence remains variable, and effectiveness depends on awareness, usability, local adaptation, and integration into routine workflows [94].

#### 3.6.2. Clinical Decision Support Systems

Clinical decision support (CDS) systems transform passive guidelines into active workflow tools by embedding appropriateness criteria into electronic ordering processes. Commonly implemented or studied frameworks include ACR Appropriateness Criteria-based systems such as ACR Select/CareSelect Imaging, ESR iGuide, RCR iRefer, and computerized physician order entry systems with integrated radiology decision support [37,40,41,55,111,112,113,114,115,116]. These systems may provide real-time recommendations regarding examination selection, alternative modalities, radiation exposure, or cost [37,114,117].

CDS systems can improve guideline concordance and referral information, but effects differ by endpoint and design [6,114,117,118]. In a stepped-wedge cluster randomized trial, the adjusted proportion of appropriate CT/MRI orders increased from 77.0% to 80.1%, without a significant change in monthly advanced-imaging ordering rates [55]. Conversely, the multicentre MIDAS cluster trial found no significant intervention effect on inappropriate requests (*p* = 0.69) [50]. An Australian before–after study reported a 9.1% reduction in CT examinations per 100 emergency presentations after electronic CDS implementation, but concurrent education and temporal changes limit causal attribution [56]. These findings distinguish improved ordering scores from reductions in utilization; neither endpoint alone proves lower total costs or better patient outcomes.

Implementation depends on referral data, coverage of clinical indications, alert burden, and clinicians’ willingness to act on recommendations [51,119,120]. For example, MIDAS encountered substantial integration and indication-matching difficulties [51]. Evidence syntheses support assessing CDS systems within broader workflow and educational strategies [114,121,122,123]. Evaluation should report how many requests can be scored, alert exposure and overrides, implementation costs, and diagnostic safety, rather than only scores among the subset successfully processed.

#### 3.6.3. Radiologist-Led Appropriateness Governance

Radiologists play a central role in imaging appropriateness, acting not only as image interpreters but also as clinical gatekeepers. Their responsibilities include justification, protocol optimization, and collaboration in clinical decision-making [43,47]. Request vetting, protocol selection, and recommendation of alternative examinations are practical mechanisms through which radiologists influence appropriateness [42].

Audit, education, and peer feedback address different mechanisms and should be judged on the outcomes actually measured. A two-cycle audit improved documentation of the clinical question, but did not demonstrate reduced imaging utilization [57]. A multicomponent general-practice intervention established feasibility and reported early experience; its protocol does not establish definitive effectiveness [124]. Peer-comparison feedback was associated with lower ordering rates in a retrospective network study, although secular change and unmeasured clinical differences remain possible explanations [125].

A 2026 systematic review of radiographer-led vetting and protocolling found mainly process-based, context-dependent evidence, with limited demonstration of shorter waiting times or fewer diagnostic delays [126]. Delegation should therefore be supported by training, explicit escalation criteria, and radiologist oversight within local professional and regulatory arrangements. Greater agreement with referral guidance should not be equated automatically with improved patient outcomes.

### 3.7. Tools to Improve Appropriateness After Imaging: Incidental Findings Management

Appropriateness does not conclude with selection of the examination; it extends into management of incidental findings. Even appropriate examinations can generate unexpected findings, while inappropriate imaging may initiate a cascade that is itself difficult to justify, exposing patients to anxiety, radiation, procedures, and additional resource use [27,28,29,38,60].

Standardized follow-up recommendations are therefore essential to reduce practice variation and prevent both over- and under-follow-up. Organ-specific guidance from the ACR Incidental Findings Committee and the Fleischner Society provides risk-stratified algorithms for pulmonary nodules, thyroid nodules, renal masses, adrenal masses, pancreatic cysts, and liver lesions [39,127,128,129,130,131].

Standardized systems must nevertheless be applied to the populations for which they were designed. For liver imaging, for example, ACR incidental-lesion guidance addresses unexpected findings in a general CT context [131], whereas the Liver Imaging Reporting and Data System (LI-RADS) is intended for patients with or at risk for hepatocellular carcinoma; both empirical evaluation of LI-RADS v2018 and its formal framework illustrate the value of explicit risk-stratified categorization in reducing interpretive ambiguity [132,133].

The wording of a report can influence downstream management; recommendations should be proportionate to patient risk and explicit about when further imaging is unnecessary [127,134]. Responsibility for communicating and completing indicated follow-up should also be assigned. In the observational Finding Incidental Disease (FIND) programme, completion of recommended follow-up increased from 30.8% to 50.7% after implementation of structured reporting and tracking [135]. This supports attention to follow-up completion, while the before–after design and substantial residual non-completion limit conclusions about clinical benefit.

Variation in guideline adherence across organ systems in elective chest CT further illustrates the implementation gap [63]. A closed-loop pathway should document both appropriate non-intervention for low-risk findings and completion of indicated assessment for clinically important findings.

### 3.8. Future Perspectives: AI-Enabled Appropriateness and Value-Based Imaging

Evidence for AI should be distinguished at four levels: technical performance on test data, external validation, prospective workflow evaluation, and demonstrated effects on patient or resource outcomes. Image analysis and reconstruction have task-specific clinical applications, but that experience cannot be transferred automatically to autonomous appropriateness decisions. Integration still requires interoperability, accountability, monitoring, and clinical supervision [8,81,82,83,84,136,137,138,139].

Large language models (LLMs) have been explored for report generation, simplification, communication, and radiological reasoning [140,141,142,143]. Studies comparing imaging suggestions with ACR criteria assess agreement in selected scenarios, not necessarily the safety or effectiveness of a live ordering pathway [144,145,146]. Prospective evaluation should address incomplete histories, comorbidity, contraindications, urgency, unequal access to alternative modalities, and performance across population groups. Reliable autonomous appropriateness assessment has not been established by these experimental comparisons.

Natural-language processing can identify incidental findings in reports, which may assist tracking [147]. Extraction accuracy must nevertheless be separated from correct risk stratification, successful communication, and completed clinically appropriate follow-up [63,135]. Report simplification studies do not establish these downstream benefits [143].

Automated auditing is another proposed use: systems could flag repeated examinations or guideline discordance for clinical review. Establishing benefit would require validation of the flags, assessment of false alerts and review burden, and comparison with existing audit workflows. Current CDS and governance studies provide a rationale for this application rather than proof of effective autonomous AI auditing [6,50,125,138].

AI does not automatically reduce workload. In an ESR survey, only 42 of 185 respondents using AI-based diagnostic algorithms (22.7%) reported a significant reduction, whereas 129 (69.8%) reported no such effect [9]. These self-reported observations are not controlled measurements of time saved, but caution against assuming that technical accuracy translates into service efficiency [136,137,138].

Technical examples illustrate both opportunities and evidential boundaries. Cross-modality brain MRI synthesis has compared paired and unpaired generative adversarial networks with diffusion and transformer approaches [148]. Bone-CNN explores a lightweight architecture for classifying primary bone tumours on radiographs [149], while a three-dimensional generative adversarial network has been evaluated for brain-tumour segmentation [150]. These dataset-based studies address image fidelity, classification, segmentation, or computational performance. They do not demonstrate fewer repeat examinations, safe substitution of synthetic images for necessary acquisitions, improved referral appropriateness, or measured net carbon savings. Synthetic image generation additionally requires evaluation for clinically consequential omissions or invented features before it can support acquisition decisions.

A scoping review published in October 2026 identified 23 studies of AI and environmental sustainability in medical imaging, but concluded that the actual environmental impact remains insufficiently established [151]. Net benefit should be measured over a defined pathway, including computation, downstream testing, and possible increases in demand, rather than inferred from model size or inference speed alone [34,99,105,106,107,108].

AI should support accountable clinical decision-making, with radiologists and referring teams responsible for contextual interpretation, exceptions, and escalation [136,137,152,153]. A tool that matches guidelines accurately still needs prospective evaluation against diagnostic safety, access, workload, and resource outcomes before routine use for appropriateness governance.

### 3.9. An Operational Framework for Clinical Practice

The proposed framework connects established tools at five decision points (Figure 1 and Table 3). At referral, the clinician documents the clinical question, urgency, relevant prior tests, and expected effect on management. Guideline-based support informs selection; uncertainty, discordance, contraindications, or unavailable alternatives trigger discussion with radiology rather than an automatic denial. When imaging is not indicated, the plan should include clinical reassessment and escalation criteria.

Radiology then selects the protocol needed for the diagnostic task and avoids unnecessary phases or repetition. Reporting should answer the clinical question and give proportionate recommendations for incidental findings, with a named team or pathway responsible for communication and follow-up. A clinically important finding that is never followed up represents a failure of appropriateness even if the original examination was justified [38,39,127,128,129,130,131,135].

For implementation, services should select a defined pathway, establish baseline measures, agree responsibilities, and introduce changes with a scheduled review. Indicators require explicit numerators, denominators, time windows, and case-mix considerations. Review should include sampled clinical assessment of cases flagged as inappropriate and of examinations deferred or declined. Reductions in activity should be accompanied by stable or improved diagnostic safety and access; emerging delays or missed diagnoses should prompt pathway reassessment. The framework is a proposal for local testing, not a validated composite score.

## 4. Limitations

This narrative review is susceptible to selection and publication bias. The initial PubMed search was a starting pool and may underrepresent health economics, implementation science, and environmental assessment. Targeted searches in additional databases, institutional sources, and citation networks broadened coverage, but were not systematic. The structured search retained its April 2026 cutoff; subsequent targeted checks may still have missed relevant recent studies.

Definitions of appropriateness and low-value imaging, populations, reference standards, and denominators vary substantially [14,36]. Referral scores, ordering rates, diagnostic yield, and patient outcomes measure different constructs. No formal risk-of-bias instrument was applied, and the selected quantitative examples should not be pooled or interpreted as representative international estimates.

Transferability depends on access, reimbursement, referral responsibilities, clinical information, infrastructure, and staffing. Before–after evaluations are vulnerable to concurrent interventions and secular trends. Environmental and lifetime-risk estimates depend on modeling assumptions and should not be treated as directly observed clinical effects [32,33,58,66,75,96]. The proposed operational framework has not undergone prospective validation, and thresholds, workload, and unintended effects require local evaluation.

Implementation remains a substantive source of uncertainty: available guidance and technically accurate tools may not be used consistently [51,111,120,121,122,123]. Evidence is especially limited for autonomous AI appropriateness assessment, sustained equity effects, and net environmental gains across complete patient pathways.

## 5. Conclusions

Imaging appropriateness requires a clinically justified examination, an appropriate protocol, and a management plan that delivers benefit to the patient. Reducing low-value activity should occur alongside improved access to necessary imaging, not through indiscriminate volume targets.

Services can translate this principle into practice by documenting the clinical question and prior imaging, embedding usable referral guidance, providing radiologist review for exceptions, and assigning responsibility for incidental findings. A small, regularly reviewed set of indicators should link referral quality and resource use to waiting times, completed follow-up, and delayed or missed diagnoses.

Future studies should compare implementation strategies prospectively and distinguish changes in appropriateness scores from utilization, total pathway costs, and patient outcomes. They should examine who gains or loses access, account for implementation burden, and measure environmental effects with explicit system boundaries and sensitivity analyses. AI tools require external and prospective validation within those same constraints.

The proposed framework makes these decisions and outcomes visible across the care pathway. Its success should be judged by safer, timely, and equitable care, together with demonstrable reductions in avoidable resource use and environmental burden.

## Figures and Tables

**Table 1:** Domains of imaging appropriateness and corresponding actions across the care pathway.

Domain	Potential Consequence	Action
Clinical value	Unhelpful testing, uncertainty, and diagnostic cascades	Clarify the question, assess expected benefit, and discuss alternatives.
Radiation protection	Avoidable exposure from unjustified or repeated examinations	Justify the examination, optimize the protocol, and avoid unnecessary follow-up.
Costs and capacity	Expenditure and displacement of clinically valuable activity	Assess pathway costs and redeploy capacity released by reduced low-value care.
Access and equity	Delayed or unavailable beneficial imaging	Monitor waiting times, modality access, and follow-up across patient groups.
Incidental findings	Over-investigation or loss of indicated follow-up	Use risk-based guidance, proportionate reporting, and assigned follow-up responsibility.
Environmental impact	Energy, consumables, travel, and downstream resource use	Avoid low-value activity and assess emissions within explicit system boundaries.
Governance and AI	Variable implementation or unsupported assumptions of benefit	Combine CDS, radiologist review, audit, and prospective evaluation of new tools.

Abbreviations: AI, artificial intelligence; CDS, clinical decision support.

**Table 2:** Selected quantitative evidence on imaging appropriateness and interventions, with limits to interpretation.

Study and Design	Reported Magnitude or Effect	Interpretation and Implementation Limits
Head CT referrals [53] Single-centre retrospective study	25% of 2498 assessable requests were usually not appropriate; 410/2908 requests were unassessable.	Clinical-information gaps affect classification. A local guideline-based estimate is not a universal overuse rate.
Imaging expenditure [54] Massachusetts claims, 2010 and 2013	Inappropriate imaging accounted for >20% of annual imaging costs for uncomplicated low-back, knee, and shoulder pain.	Selected commercially insured population; claims-based appropriateness and restricted indications. Expenditure is not equivalent to recoverable cash savings.
Ambulatory CDS [55] Stepped-wedge cluster randomized trial	22,279 eligible orders. Adjusted appropriate-order proportion: 77.0% before versus 80.1% after alerts; no significant change in monthly ordering rates.	A score improvement does not establish reduced utilization. Requires structured indications and usable alerts.
Hospital CDS [50] MIDAS cluster randomized trial	26 departments, 3 hospitals; 65,764 scored requests. No significant between-group effect on inappropriate requests (*p* = 0.69).	Low baseline inappropriateness and indication coverage constrain impact and generalizability. Integration and adoption must be assessed.
ED electronic CDS [56] Retrospective before–after study	About 7% of orders modified; CT examinations per 100 ED presentations decreased by 9.1%.	Concurrent education and temporal effects limit causal attribution. Changes in clinical outcomes and total pathway costs remain separate questions.
Referral documentation [57] Two-cycle audit, 50 forms per cycle	A documented clinical question increased from 3/50 (6%) to 41/50 (82%) after a standardized form and education.	Small uncontrolled audit of documentation. Fewer scans or better patient outcomes were not demonstrated.
Imaging footprint [33] Life cycle assessment in two Australian services	Average MRI: 17.5 kg CO_2_e/scan; CT: 9.2. Additional-scan estimates: approximately 1.1 kg for each, excluding standby emissions.	Average and incremental impacts answer different questions. Electricity mix, equipment, utilization, and boundaries limit transferability.
Inappropriate-imaging emissions [58] US Medicare Part B model, 2017–2021	Assumed 4–26% inappropriateness; average annual estimate 3.55–129.2 kilotonnes CO_2_e across MRI, CT, radiography, and ultrasound.	Scenario bounds, not a confidence interval. Combines utilization, external inappropriateness estimates, and emission factors; no patient-level adjudication.

Abbreviations: CDS, clinical decision support; CT, computed tomography; ED, emergency department; MRI, magnetic resonance imaging; CO_2_e, carbon dioxide equivalent. Estimates retain their original populations and denominators. Rows are illustrative and are not a pooled synthesis. Before–after associations do not establish causality; modeled emissions are not directly measured avoided emissions.

**Table 3:** Proposed responsibilities and measurable indicators across the imaging pathway.

Decision Point and Responsibility	Process or Outcome Indicator	Balancing Measure
Justify the request Referrer; radiologist for exceptions	Requests with a clear clinical question/requests audited; guideline concordance among assessable requests, with unassessable requests reported separately.	Subsequent urgent imaging, delayed diagnoses, or unplanned return visits after deferral.
Select modality and protocol Radiologist and radiographer	Requests revised after review/requests reviewed; avoidable repeat or additional-phase examinations; dose for the defined diagnostic task.	Diagnostic adequacy and time to definitive investigation, including access to alternatives.
Report and manage findings Reporting radiologist and responsible clinical team	Actionable findings with a clear recommendation and responsible team/actionable findings; indicated follow-up completed within the agreed interval.	Unnecessary follow-up of low-risk findings and clinically important findings lost to follow-up.
Evaluate service effects Clinical and radiology governance team	Imaging per defined clinical episode; waiting times by urgency; pathway costs; energy or CO_2_e per defined activity and system boundary.	Missed or delayed diagnoses, clinician workload, patient experience, and unequal access by geography or socioeconomic group.
Review and adapt Multidisciplinary governance team	Documented review of audit results and exceptions; completion of agreed corrective actions.	Unintended denial of justified imaging and deterioration in safety or follow-up completion.

Indicators are proposed for local implementation and validation. Balancing measures assess unintended harm; thresholds and review intervals should reflect the clinical pathway. Rates should use defined eligible populations and be stratified by relevant access and equity variables where feasible.

## Data Availability

No patient-level data were collected. The published sources supporting the synthesis are identified in the reference list.
